# Supplementary material for: PRC2-AgeIndex as a universal biomarker of aging and rejuvenation
Source: Nat Commun. 2024 Jul 16;15:5956. doi: 10.1038/s41467-024-50098-2 (PMC11250797; doi:10.1038/s41467-024-50098-2)
Supplement: Supplementary file 1 — Supplementary Information [file 41467_2024_50098_MOESM1_ESM.pdf]

Supplementaty information file

Supplementaty Figure 1

Supplementaty Figure 2

Supplementary figure 1

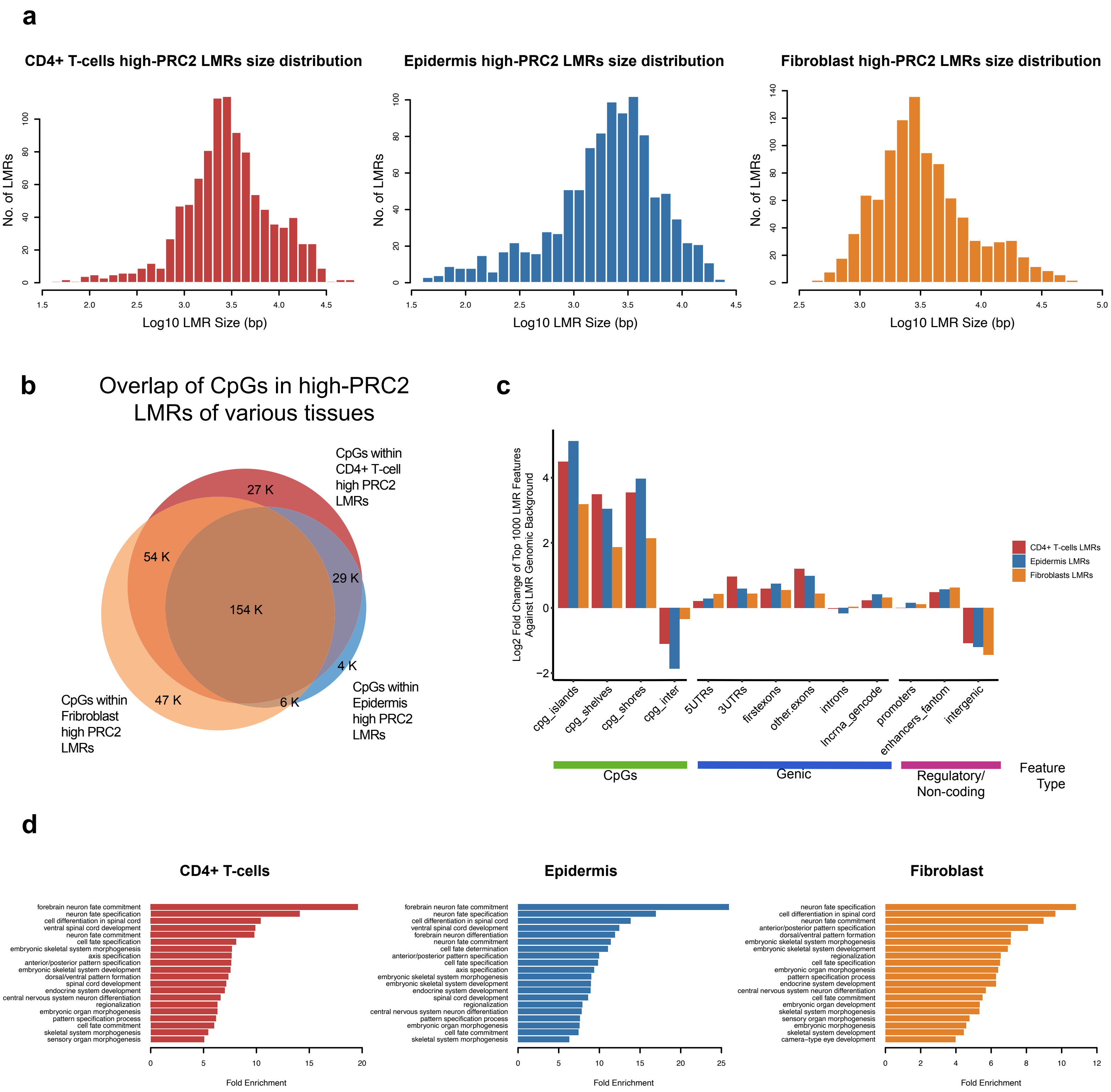

Supplementary Figure 1: a. Distribution of top 1000 PRC2-binding LMR size (bp) in CD4+ T cells (left panel), epidermis (middle panel) and passaged fibroblasts (right panel). **b.** Venn diagram of overlapping CpGs between top 1000 PRC2-binding LMRs in CD4+ T cells, epidermis and passaged fibroblasts. **c.** Log2 fold change of genomic annotation proportions in top 1000 PRC2-binding LMRs in CD4+ T cells, epidermis and passaged fibroblasts compared to respective genomic proportions in all LMRs (as background) for a given tissue (see Table S2 for full list of gene and feature annotations for the top 1000 LMRs and Methods for more detail). **d.** GREAT Gene Ontology analysis of promoters, enhancers and first exons in top 1000 PRC2-binding LMRs in CD4+ T cells (left panel), epidermis (middle panel) and passaged fibroblasts (right panel).

Supplementary figure 2

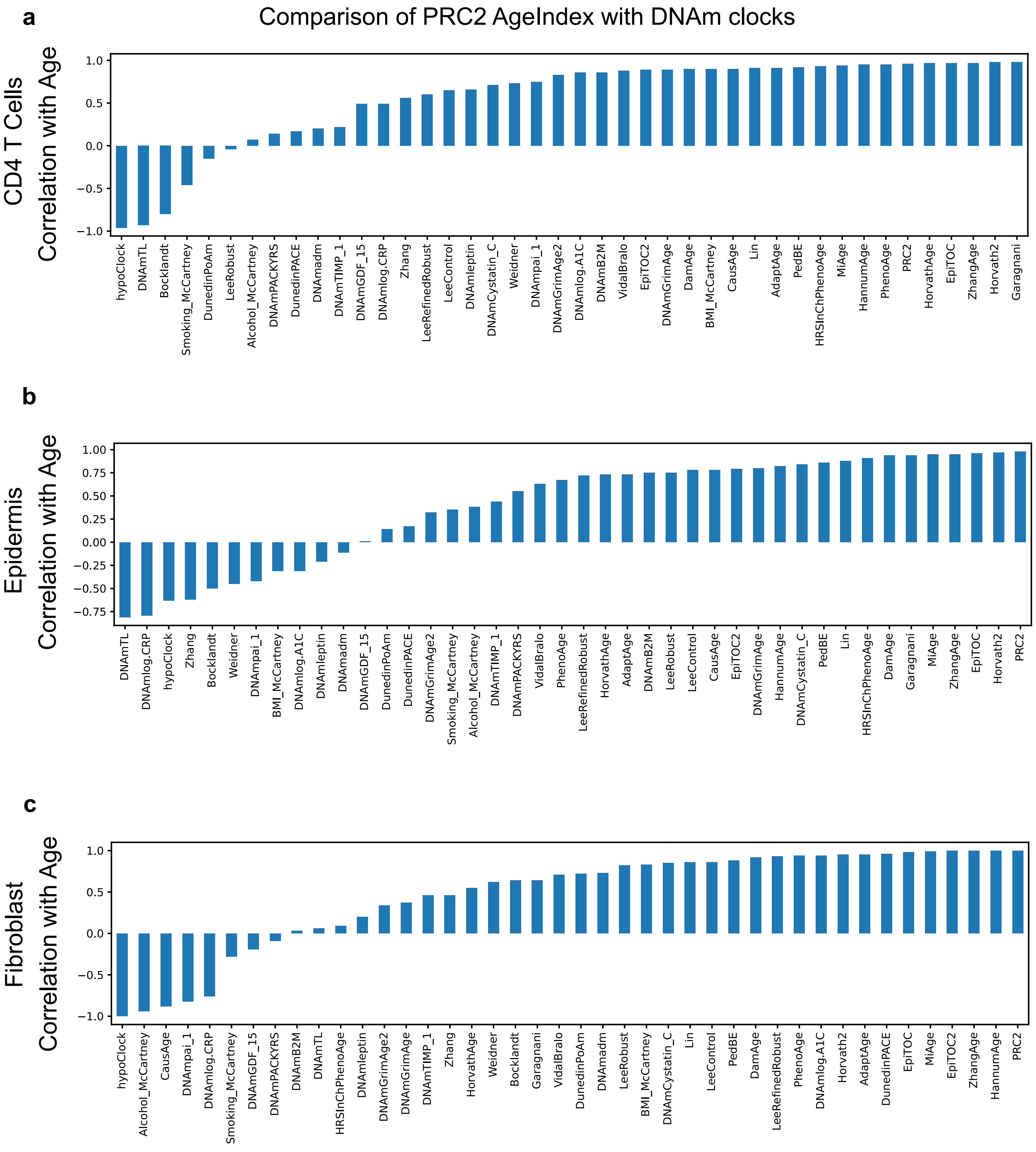

Supplementary Figure 2: Comparison of Pearson correlation of the PRC2 index with 41 other DNAm-based age/health predictors, applied to CD4 T-cells (**a**), epidermis (**b**) and fibroblasts (**c**).
