# Supplementary material for: PRC2-AgeIndex as a universal biomarker of aging and rejuvenation
Source: Nat Commun. 2024 Jul 16;15:5956. doi: 10.1038/s41467-024-50098-2 (PMC11250797; doi:10.1038/s41467-024-50098-2)
Supplement: Supplementary file 3 — Description of Additional Supplementary Files [file 41467_2024_50098_MOESM3_ESM.pdf]

Description of Additional Supplementary Files

Supplementary Data 1: List of publicly available datasets used in the manuscript for different analyses
