# Supplementary material for: PRC2-AgeIndex as a universal biomarker of aging and rejuvenation
Source: Nat Commun. 2024 Jul 16;15:5956. doi: 10.1038/s41467-024-50098-2 (PMC11250797; doi:10.1038/s41467-024-50098-2)
Supplement: Supplementary file 5 — Reporting Summary [file 41467_2024_50098_MOESM5_ESM.pdf]

nature portfolio

Corresponding author(s): Vittorio Sebastiano

Last updated by author(s): Jun 20, 2024

Reporting Summary

Nature Portfolio wishes to improve the reproducibility of the work that we publish. This form provides structure for consistency and transparency in reporting. For further information on Nature Portfolio policies, see our Editorial Policies and the Editorial Policy Checklist.

Statistics

For all statistical analyses, confirm that the following items are present in the figure legend, table legend, main text, or Methods section.

n/a	Confirmed	
☐	☒	The exact sample size (n) for each experimental group/condition, given as a discrete number and unit of measurement
☐	☒	A statement on whether measurements were taken from distinct samples or whether the same sample was measured repeatedly
☐	☒	The statistical test(s) used AND whether they are one- or two-sided<br>*Only common tests should be described solely by name; describe more complex techniques in the Methods section.*
☒	☐	A description of all covariates tested
☒	☐	A description of any assumptions or corrections, such as tests of normality and adjustment for multiple comparisons
☐	☒	A full description of the statistical parameters including central tendency (e.g. means) or other basic estimates (e.g. regression coefficient) AND variation (e.g. standard deviation) or associated estimates of uncertainty (e.g. confidence intervals)
☐	☒	For null hypothesis testing, the test statistic (e.g. F, t, r) with confidence intervals, effect sizes, degrees of freedom and P value noted<br>*Give P values as exact values whenever suitable.*
☒	☐	For Bayesian analysis, information on the choice of priors and Markov chain Monte Carlo settings
☒	☐	For hierarchical and complex designs, identification of the appropriate level for tests and full reporting of outcomes
☒	☐	Estimates of effect sizes (e.g. Cohen's d, Pearson's r), indicating how they were calculated

Our web collection on statistics for biologists contains articles on many of the points above.

Software and code

Policy information about availability of computer code

Data collection: The data used for these studies have been selected from GEO database, and the identification number for each experiment is reported in Source Data File 1

Data analysis: All of our scripts to analyze the WGBS data, produce the figures, and generate the final PRC2 LMRs are freely accessible on Github at https://github.com/moqri/PRC2-AgeIndex

For manuscripts utilizing custom algorithms or software that are central to the research but not yet described in published literature, software must be made available to editors and reviewers. We strongly encourage code deposition in a community repository (e.g. GitHub). See the Nature Portfolio guidelines for submitting code & software for further information.

Data

Policy information about availability of data

All manuscripts must include a data availability statement. This statement should provide the following information, where applicable:

- Accession codes, unique identifiers, or web links for publicly available datasets
- A description of any restrictions on data availability
- For clinical datasets or third party data, please ensure that the statement adheres to our policy

GSE52972 Human Epidermis 6 18 to 83 years WGBS 1, 2 0 https://www.ncbi.nlm.nih.gov/geo/query/acc.cgi?acc=GSE52972
GSE31263 Human CD4+ T cells 2 1 neonatal and 1 centenerian WGBS 1,2,6 0 https://www.ncbi.nlm.nih.gov/geo/query/acc.cgi?acc=GSE31263

March 2021

GSM2472741 - SRX2528911 Mouse mESCs 1 N/A ChIP-seq Ezh2 rep1 2,3,4 0 https://www.ncbi.nlm.nih.gov/geo/query/acc.cgi?acc=GSM2472741
GSM2472742 - SRX2528912 Mouse mESCs 1 N/A ChIP-seq Ezh2 rep2 2,3,4 0 https://www.ncbi.nlm.nih.gov/geo/query/acc.cgi?acc=GSM2472742
GSM3243624 - SRX4338248 Mouse mESCs 1 N/A ChIP-seq Suz12 2,3,4 0 https://www.ncbi.nlm.nih.gov/geo/query/acc.cgi?acc=GSM3243624
ENCSR000ASY - ENCFF105JFX Human hESCs, H1 1 N/A ChIP-seq EZH2 - hg38 1,2,3,5,6 0 https://www.encodeproject.org/files/ENCFF105JFX/
ENCSR000ATS - ENCFF224RZW Human hESCs, H1 1 N/A ChIP-seq SUZ12 - hg38 1,2,3,5,6 0 https://www.encodeproject.org/files/ENCFF224RZW/
ENCSR000ASY - ENCFF000AVT Human hESCs, H2 1 N/A ChIP-seq EZH2 - hg19 1,2,3,5,6 0 https://www.encodeproject.org/files/ENCFF000AVT/
ENCSR000ATS - ENCFF027VOM Human hESCs, H3 1 N/A ChIP-seq SUZ12 - hg19 1,2,3,5,6 0 https://www.encodeproject.org/files/ENCFF027VOM/
GSE16256 Human hESC, H1 1 N/A WGBS   1 0 https://www.ncbi.nlm.nih.gov/geo/query/acc.cgi?acc=GSE16256
GSE79798 Human CD4+ T cells 6 18 to 86 years old WGBS   1,2,6 1 https://www.ncbi.nlm.nih.gov/geo/query/acc.cgi?acc=GSE79798
GSE89274 Mouse Hepatocytes 16 4 2-months and 12 22-months WGBS   2, 4 0 https://www.ncbi.nlm.nih.gov/geo/query/acc.cgi?acc=GSE89274
GSE152027 Human PMBC 656 19 to 101 years Methyl450   3 0 https://www.ncbi.nlm.nih.gov/geo/query/acc.cgi?acc=GSE152027
GSE40279 Human PMBC 689 18 to 70 years Methyl450   3 0 https://www.ncbi.nlm.nih.gov/geo/query/acc.cgi?acc=GSE40279
GSE42861 Human PMBC 471 13 to 72 years Methyl450   3 0 https://www.ncbi.nlm.nih.gov/geo/query/acc.cgi?acc=%20GSE42861
GSE55763 Human PMBC 2711 23 to 75 years Methyl450   3 0 https://www.ncbi.nlm.nih.gov/geo/query/acc.cgi?acc=GSE55763
GSE120137 Mouse "Adipose, Kidney,
Liver, Lung, Blood " 40 20 2-months and 20 20-months RRBS   3 1 https://www.ncbi.nlm.nih.gov/geo/query/acc.cgi?acc=GSE120137
SRP069120 Mouse Hepatocytes 20 4-months and 26-months scWGBS   3 2 https://www.ncbi.nlm.nih.gov/Traces/study/?acc=SRP069120
GSE80672 Mouse Blood 110 3 to 35 months RRBS   4 0 https://www.ncbi.nlm.nih.gov/geo/query/acc.cgi?acc=GSE80672
GSE190665 Mouse Skin 12 7 old and 5 treated old MethyEpic   4 0 https://www.ncbi.nlm.nih.gov/geo/query/acc.cgi?acc=GSE190665
TCGA Human Different tissues 40 Different ages, tumor vs adjecant normal WGBS   5 0 https://www.cancer.gov/about-nci/organization/ccg/research/structural-genomics/tcga
GSE107729 Human Oligocytes 6 3 under 40 and 3 ablove 70 years WGBS   5 0 https://www.ncbi.nlm.nih.gov/geo/query/acc.cgi?acc=GSE107729
GSE79798 Human Fibroblasts 1 Fetal, passage 4 to 33 WGBS   5 0 https://www.ncbi.nlm.nih.gov/geo/query/acc.cgi?acc=GSE79798
GSE253987 - GSE253985 Human Fibroblasts 6 Neonatal (passage 2, 4 and 8) and old (>80, passage 2, 5 and 8) WGBS   6 0 https://www.ncbi.nlm.nih.gov/geo/query/acc.cgi?acc=GSE253985
GSE253987 - GSE253773 Human Fibroblasts 5 Neonatal (two replicates), old (three replicates) ChIP-seq   6 0 https://www.ncbi.nlm.nih.gov/geo/query/acc.cgi?acc=GSE253773
GSE253987 - GSE253773 Human CD4+ T cells 3 Three donors aged 23, 17 and 17 ChIP-seq   6 0 https://www.ncbi.nlm.nih.gov/geo/query/acc.cgi?acc=GSE253773
ENCFF918OQM Human CD4+ T cells 1 Input file for CD4+ T-cells ChIP-seq   6 0 https://www.encodeproject.org/files/ENCFF918OQM/

Human research participants

Policy information about studies involving human research participants and Sex and Gender in Research.

Reporting on sex and gender	N/A
Population characteristics	N/A
Recruitment	N/A
Ethics oversight	N/A

Note that full information on the approval of the study protocol must also be provided in the manuscript.

Field-specific reporting

Please select the one below that is the best fit for your research. If you are not sure, read the appropriate sections before making your selection.

[x] Life sciences   [ ] Behavioural & social sciences   [ ] Ecological, evolutionary & environmental sciences

For a reference copy of the document with all sections, see nature.com/documents/nr-reporting-summary-flat.pdf

Life sciences study design

All studies must disclose on these points even when the disclosure is negative.

Sample size	N/A
Data exclusions	N/A
Replication	N/A
Randomization	N/A
Blinding	N/A

Reporting for specific materials, systems and methods

We require information from authors about some types of materials, experimental systems and methods used in many studies. Here, indicate whether each material, system or method listed is relevant to your study. If you are not sure if a list item applies to your research, read the appropriate section before selecting a response.

Materials & experimental systems

n/a	Involved in the study
☐	☒ Antibodies
☐	☒ Eukaryotic cell lines
☒	☐ Palaeontology and archaeology
☒	☐ Animals and other organisms
☒	☐ Clinical data
☒	☐ Dual use research of concern

Methods

n/a	Involved in the study
☐	☒ ChIP-seq
☒	☐ Flow cytometry
☒	☐ MRI-based neuroimaging

Antibodies

Antibodies used	EZH2, Cell Signaling cat n. 5246
Validation	The antibody is ChIP-grade and was used in other publications and validated using ChIP followed by qPCR prior to sequencing.

Eukaryotic cell lines

Policy information about cell lines and Sex and Gender in Research

Cell line source(s)	Fibroblast primary cells were obtained from cadavers (old) or from foreskin removal surgeries (neonatal)
Authentication	None of the cell lines used in the study were authenticated
Mycoplasma contamination	Cell lines have been tested for Mycoplasma contamination prior to use and they were negative
Commonly misidentified lines (See ICLAC register)	N/A

ChIP-seq

Data deposition

☒ Confirm that both raw and final processed data have been deposited in a public database such as GEO.

☒ Confirm that you have deposited or provided access to graph files (e.g. BED files) for the called peaks.

Data access links<br>*May remain private before publication.*	https://www.ncbi.nlm.nih.gov/geo/query/acc.cgi?acc=GSE253773
Files in database submission	NEO_P2_Merged_fe.bw<br>OLD_P2_Merged_fe.bw<br>EZH2_CD4_pooled_ENCinp_fe.bw<br>N1P2_peaks.narrowPeak<br>N2P2_peaks.narrowPeak<br>O1P2_peaks.narrowPeak<br>O2P2_peaks.narrowPeak<br>O3P2_peaks.narrowPeak<br>N1P2_peaks.narrowPeak<br>N2P2_peaks.narrowPeak<br>O1P2_peaks.narrowPeak<br>O2P2_peaks.narrowPeak<br>O3P2_peaks.narrowPeak<br>Inp_N1P2_1.fq.gz<br>Inp_N2P2_1.fq.gz<br>Inp_O1P2_1.fq.gz<br>Inp_O2P2_1.fq.gz<br>Inp_O3P2_1.fq.gz<br>IP_N1P2_1.fq.gz<br>IP_N2P2_1.fq.gz

IP_O1P2_1.fq.gz
IP_O2P2_1.fq.gz
IP_O3P2_1.fq.gz
EZH2_CD4_d32_1.fq.gz
EZH2_CD4_d43_1.fq.gz
EZH2_CD4_d48_1.fq.gz
Inp_N1P2_2.fq.gz
Inp_N2P2_2.fq.gz
Inp_O1P2_2.fq.gz
Inp_O2P2_2.fq.gz
Inp_O3P2_2.fq.gz
IP_N1P2_2.fq.gz
IP_N2P2_2.fq.gz
IP_O1P2_2.fq.gz
IP_O2P2_2.fq.gz
IP_O3P2_2.fq.gz
EZH2_CD4_d32_2.fq.gz
EZH2_CD4_d43_2.fq.gz
EZH2_CD4_d48_2.fq.gz

Genome browser session
(e.g. UCSC)

https://genome.ucsc.edu/cgi-bin/hgTracks?db=hg38&lastVirtModeType=default&lastVirtModeExtraState=&virtModeType=default&virtMode=0&nonVirtPosition=&position=chr22%3A17368052%2D17372061&hgsid=2189213828_OqWXN5pQD2moaJTX2czaxiDl4ckH

Methodology

Replicates

Two biological replicates were used for the neonatal fibroblast samples and three were used for both old fibroblasts and CD4 T-cells.

Sequencing depth

Sample total_reads paired_aligned_uniquely sequencing
IP_N1P1 21138445 13340158 150bp reads paired end
IP_N2P1 21987271 14095245 150bp reads paired end
IP_O1P1 20950043 14692545 150bp reads paired end
IP_O2P1 21471081 13750082 150bp reads paired end
IP_O3P1 19686906 12982416 150bp reads paired end
Inp_N1P1 28538643 19853471 150bp reads paired end
Inp_N1P10 25033079 17139740 150bp reads paired end
Inp_N2P1 26511358 17570735 150bp reads paired end
EZH2_CD4_d32 29661602 15312960 150bp reads paired end
EZH2_CD4_d43 29113592 16859093 150bp reads paired end
EZH2_CD4_d48 30239961 19554437 150bp reads paired end

Antibodies

EZH2, Cell Signaling cat n. 5246

Peak calling parameters

```
#mapping
bowtie2 -X2000 -x $bwt2_idx -1 ../Trimmed/${fileID}_1_val_1.fq.gz -2 ../Trimmed/${fileID}_2_val_2.fq.gz 2> $f.log -p 18 -S ./${f}.sam

#peakcalling
macs2 callpeak -t ../mapping_filtering/IP_$fileID.dedup \
        -c ../mapping_filtering/Inp_$fileID.dedup \
        -g hs -p 1e-2 --keep-dup all -B --nomodel --extsize $FRAG --SPMR \
        -n ${fileID} \
        --outdir . \
```

Data quality

For peakcalling, p < 0.01 was set. Peak calling was called on neonatal fibroblasts, old fibroblasts and CD4 T-cells merged by replicate.
No of peaks over >= FDR 5% and FC >=5 for merged samples:
Sample NoPeaks
NEO_P1_Merged_peaks.narrowPeak 3180
OLD_P1_Merged_peaks.narrowPeak 3038
EZH2_CD4_pooled_ENCinp_peaks.narrowPeak 4019

Narrowpeak files for individual samples was only called for neonatal and old fibroblasts as these samples were taken forward for diffbind analysis and individual (rather than merged samples) were required.
No of peaks over >= FDR 5% and FC >=5 for individual samples:
Sample NoPeaks
N1P1_peaks.narrowPeak 2852
N2P1_peaks.narrowPeak 3295
O1P1_peaks.narrowPeak 2983
O2P1_peaks.narrowPeak 3505
O3P1_peaks.narrowPeak 2428

For primary analysis for our PRC2-AgeIndex, fold enrichment was calculated for each merged sample ($p < 0.00001$) using macs2 bdgcmp as follows:

macs2 bdgcmp -t ${fileID}_treat_pileup.bdg -c ${fileID}_control_lambda.bdg -o ${fileID}_fe.bdg -m FE -p 0.00001

The output was saved as a bigwig file and used to order low methylated regions (LMRs) by fold enrichment.

Software

Bowtie2 was used to map ChIP fastq files. MACS2 was used for peakcalling and fold enrichment calculation. Low methylated regions were sorted by EZH2 fold enrichment using the following custom scripts:

https://github.com/moqri/PRC2-AgeIndex/blob/main/PRC2-AgeIndex_Manuscript_Figures/LMR_PRC2_Binding.ipynb

https://github.com/moqri/PRC2-AgeIndex/blob/main/PRC2-AgeIndex_Manuscript_Figures/Manuscript_Figures_PRC2-AgeIndex.ipynb
